# Biotransformation of Phenolics in Spent Liquor from Aqueous Ammonia Pretreatment

**DOI:** 10.1002/cssc.202500881

**Published:** 2025-09-10

**Authors:** Shengfei Zhou, Maximiliano García‐Mancilla, Jordi Francis Clar, Troy M. Runge, Timothy J. Donohue, Daniel R. Noguera, Steven D. Karlen

**Affiliations:** ^1^ Great Lakes Bioenergy Research Center Wisconsin Energy Institute University of Wisconsin‐Madison Madison WI 53706 USA; ^2^ Department of Bacteriology University of Wisconsin‐Madison Madison WI 53706 USA; ^3^ Department of Biological Systems Engineering University of Wisconsin‐Madison Madison WI 53706 USA; ^4^ Department of Civil and Environmental Engineering University of Wisconsin‐Madison Madison WI 53706 USA

**Keywords:** biomass, biotransformation, phenol, phenolic amides, renewable chemicals

## Abstract

Spent liquors of biomass pretreatment provide a source for renewable chemical production. These liquors require treatment before being discharged; otherwise, they negatively impact the environment. Herein, spent liquors from aqueous ammonia pretreatment of poplar wood are characterized for phenolic content via liquid chromatography–mass spectrometry and nuclear magnetic resonance spectroscopy. The main phenolics are phenol, *p*‐hydroxybenzamide (*p*HBAm), and *p*‐hydroxybenzoic acid (*p*HBA), of which *p*HBAm and *p*HBA are produced from the ester‐linked *p*‐hydroxybenzoates in poplar wood. Phenol is produced from *p*HBA via decarboxylation. The potential biotransformation of the extracted phenolics into 2‐pyrone‐4,6‐dicarboxylic acid (PDC) is assessed using an engineered strain of *Novosphingobium aromaticivorans* DSM12444 (PDC strain). Biotransformation of *p*HBAm to PDC is shown to be possible in the presence of *p*HBA, but not when *p*HBAm is the sole phenolic substrate, this is the first reported observation of *N. aromaticivorans* producing PDC from an aromatic amide. The phenol present is not transformed to PDC and does not inhibit PDC production. This study demonstrates that the phenolic amide in spent liquor from ammonia pretreatment can be valorized via biotransformation using *N. aromaticivorans*, which adds to the growing versatility of *N. aromaticivorans* as a microbial chassis for converting plant‐derived compounds to useful products.

## Graphical abstract

Aqueous ammonia pretreatment of poplar releases *p*‐hydroxybenzamide (*p*HBAm) and *p*‐hydroxybenzoic acid (*p*HBA) into the spent liquor and converts some of the *p*HBA to phenol. An engineered strain of *N. aromaticivorans* transforms *p*HBAm and *p*HBA into the bioplastic precurso 2‐pyrone‐4,6‐dicarboxylic acid. These findings expand the versatility of *N. aromaticivorans* as a microbial chassis for converting plant‐derived compounds to useful products.

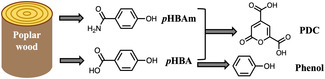

## 1 Introduction

Strategies to produce materials, chemicals, and fuels from renewable resources like biomass need to be developed to promote the bioeconomy and reduce long‐term climate changes due to unrestricted use of fossil fuels. Pretreatment of biomass can alter the cell wall structure, extract minerals, (partially) remove lignin, hemicellulose, and microbial toxins, and facilitate downstream steps of deconstruction to recover the carbohydrates stored in cellulosic polymers. These alterations combine to unlock the potential of biomass and make pretreatment an essential step for biomass conversion to biofuels and renewable chemicals. Various biomass pretreatment approaches have been studied with focus on the effects of pretreatment on biomass properties and biomass‐to‐biofuel conversion (e.g., bioethanol,^[^ ^1^ ^]^ *n*‐butanol and isobutanol,^[^ ^2^ ^]^ and biodiesel)^[^ ^3^, ^4^ ^]^, improving and optimizing sugar (e.g., glucose and xylose) yields at enzymatic digestion, and the determination and subsequent removal of inhibitors to microbial fermentations.^[^ ^1^, ^5^, ^6^ ^]^ Ammonia pretreatment, a widely studied pretreatment, is known to alter and remove lignin, decrystallize cellulose, and increase accessible surface area in biomass.^[^ ^7^ ^]^

Biomass pretreatment usually generates a stream of spent liquor with dissolved carbohydrates and aromatics. For instance, the spent liquors from acidic sulfite pretreatment (SPORL) can dissolve up to 25 wt% of the original solids in wood.^[^ ^8^ ^]^ An aqueous ammonia pretreatment of energy cane bagasse can remove 26–44 wt% of the original solids.^[^ ^9^ ^]^ Thus, the spent liquor needs to be properly treated before being released into environment. Researchers have explored using spent liquor for subsequent biomass pretreatment and fermentation medium preparation, which can save water, chemicals, and energy.^[^ ^10^, ^11^, ^12^, ^13^, ^14^, ^15^, ^16^ ^]^ Another strategy is to use the organics in spent liquor to make other valuable chemicals. Spent liquor from various processes has been converted to polyhydroxyalkanoates, biodiesel, hydrogen, biogas, organic acids (e.g., succinic acid), aromatic compounds (e.g., catechol and aromatic hydrocarbons), and bio‐oil biologically or thermochemically, for applications in fuels, chemicals, and polymers.^[^ ^3^ ^]^ The valorization of spent liquor after ammonia pretreatment is less studied, though the dissolved aromatics in spent liquor could be valuable feedstock for making platform chemicals, polymers, and pharmaceuticals.

However, these dissolved phenolics, often referred as “lignin fragments”,^[^ ^3^ ^]^ are not well characterized due to the low value and chemical complexity of spent liquor. Since the ester‐linked *p*‐hydroxybenzoate (*p*HB‐ester) exists in natural lignins of poplars, willows, and palms,^[^ ^17^, ^18^, ^19^, ^20^, ^21^ ^]^ *p*‐hydroxybenzoic acid (*p*HBA) can be produced from it using mild alkaline conditions.^[^ ^22^, ^23^, ^24^ ^]^ During the aqueous ammonia pretreatment of poplar wood at elevated pretreatment temperatures (>100 °C), the lignin‐bound *p*HB‐ester preferentially forms *p*‐hydroxybenzamide (*p*HBAm) over hydrolysis of the ester bond to form *p*HBA,^[^ ^24^ ^]^ then *p*HBA can be further degraded to phenol via decarboxylation. Phenol has been reported to be in the black liquor from alkaline pulping (165–170 °C) of mixed beech, poplar, oak, and chestnut wood.^[^ ^25^, ^26^ ^]^ Phenol was also synthesized from technical lignin via alkaline hydrothermal processing at 250–400 °C.^[^ ^26^, ^27^, ^28^ ^]^ To better understand the spent liquor from ammonia pretreatment of poplar wood and utilize it, detailed characterization of the dissolved aromatics will be needed.

Metals have been used as catalysts for lignin degradation, spent liquor treatment, and biomass pretreatment. Heavy metals have been known to accelerate the alkaline hydrogen peroxide oxidation of lignin models for decades.^[^ ^29^, ^30^ ^]^ The poly‐phenols in olive oil mill wastewater can be almost completely (99%) removed by the copper‐catalyzed alkaline hydrogen peroxide oxidation.^[^ ^31^ ^]^ The copper(II) 2,2′‐bipyridine catalyzed alkaline hydrogen peroxide (Cu‐AHP) pretreatment is found to greatly improve the monosaccharides yields of subsequent enzymatic hydrolysis of plant cell wall for all biomass types.^[^ ^32^ ^]^ Metals (e.g., Na,^[^ ^33^ ^]^ Li,^[^ ^34^ ^]^ Ba,^[^ ^35^ ^]^ Cu,^[^ ^36^, ^37^ ^]^ Pd,^[^ ^38^, ^39^ ^]^ Au,^[^ ^40^ ^]^ Ag,^[^ ^41^ ^]^ Rh,^[^ ^42^ ^]^ and Fe/Ru)^[^ ^43^ ^]^ can also catalyze the decarboxylation of carboxylic acids under a wide range of conditions. In this sense, the effects of metals on ammonia pretreatment are of great interests.

After pretreatment, biotransformation of lignocellulosic biomass to biofuels (e.g., bioethanol,^[^ ^1^ ^]^ *n*‐butanol and isobutanol,^[^ ^2^ ^]^ and biodiesel)^[^ ^3^, ^4^ ^]^ and platform chemicals (e.g., polyhydroxyalkanoates^[^ ^3^ ^]^ and *cis,cis*‐muconic acid)^[^ ^44^ ^]^ provides accessible pathways to bioeconomy. The advantages of biotransformation over chemical conversion usually include using aqueous systems for the reaction, lower operating temperature and pressure, and operating under physically and environmentally safer conditions. One such biotransformation platform converts biomass lignin‐derived phenolics to 2‐pyrone‐4,6‐dicarboxylic acid (PDC), a potential polymer precursor for bioplastics and epoxy adhesives, which was originally pioneered by Masai et al.^[^ ^45^, ^46^, ^47^, ^48^ ^]^ PDC production from biomass streams has been studied in several microbial chassis, including *Pseudomonas* species,^[^ ^49^, ^50^, ^51^, ^52^ ^]^ *Sphingomonas paucimobilis* SYK‐6,^[^ ^45^, ^46^ ^]^ *Escherichia coli*,^[^ ^53^, ^54^ ^]^ and *Novosphingobium aromaticivorans* PDC strain.^[^ ^55^, ^56^, ^57^ ^]^ Of these chassis, the *N. aromaticivorans* PDC strain naturally encodes the enzymes to funnel the three major types of lignin derived phenolics, i.e., sinapyl (S), coniferyl (G), and *p*‐coumaryl (H) derivatives to PDC,^[^ ^55^, ^56^, ^57^ ^]^ making it a research tool capable of quantifying the phenolics that could be converted to PDC from various streams derived from biomass processing, like the spent liquor from aqueous ammonia pretreatment.

In the present study, poplar wood chips were pretreated with aqueous ammonia, and the effects of metal additives were tested. The phenolics in spent liquor were extracted using ethyl acetate and the main phenolics were identified to be phenol, *p*HBAm, and *p*HBA. Biotransformation of these phenolics using *N. aromaticivorans* PDC strain showed that *p*HBAm and *p*HBA were successfully converted into PDC, making phenolic amides a new feedstock for the *N. aromaticivorans* platform. Phenol was not converted to PDC by *N. aromaticivorans* and did not inhibit the biotransformation of *p*HBAm and *p*HBA to PDC.

## 2 Experimental Section

### 2.1 Materials and Chemicals

Poplar wood chips (air dried, ≈5 mm particle size, debarked NM6 poplar, harvested in 2018) were provided by the Great Lakes Bioenergy Research Center (GLBRC) at the University of Wisconsin‐Madison (UW‐Madison). The 2‐pyrone‐4,6‐dicarboxylic acid (PDC) standard was biologically synthesized from *p*HBA as described earlier^[^ ^58^ ^]^ using a engineered strain of *Novosphingobium aromaticivorans* DSM12444 (PDC strain) to accumulate PDC when transforming plant‐derived phenolics.^[^ ^55^ ^]^ The PDC strain has deletions in *ligI* (Saro_2819 or SARO_RS14300 in the most recent annotation), *desC* (Saro_2864 or SARO_RS14525), and *desD* (Saro_2865 or SARO_RS14530) allowing it to extracellularly accumulate PDC, an intermediate of phenolics metabolism. Reverse osmosis (RO) water was used if not noted.

Other chemicals used in this study include ammonia hydroxide solution (28.0–30.0% NH_3_ basis, Sigma‐Aldrich), copper(II) sulfate (CuSO_4_, ≥ 99%, Sigma‐Aldrich), 2,2'‐bipyridine (bpy, ACS grade, Sigma‐Aldrich), palladium(II) acetate (Pd(OAc)_2_, 98%, Aldrich), aluminum oxide (Al_2_O_3_, type WN‐6, neutral, activity grade super I, Sigma‐Aldrich), nickel(II) chloride hexahydrate (NiCl_2_·6H_2_O, 99.9999%, Aldrich), magnesium acetate tetrahydrate (Mg(OAc)_2_·4H_2_O, 99%, Aldrich), ethanol (anhydrous, 200 proof, Decon Labs), sulfuric acid 72 wt% (H_2_SO_4_, RICCA), ethyl acetate (LC–MS CHROMASOLV, Fluka Analytical), dimethyl sulfoxide‐d_6_ (DMSO‐d_6_, 99.9 atom% D, Sigma‐Aldrich), dimethyl sulfoxide (DMSO, certified ACS, Fisher Chemical), formic acid (LC/MS, Fisher Scientific), acetonitrile (ACN, CHROMASOLV LC–MS, Honeywell), methanol (MeOH, CHROMASOLV LC–MS, Honeywell), sodium hydroxide (NaOH, ≥97.0%, Sigma‐Aldrich), 4‐hydroxybenzamide (*p*HBAm, 98%, Aldrich), 4‐hydroxybenzoic acid (*p*HBA, 99%, Aldrich), and phenol (≥99%, Sigma‐Aldrich).

### 2.2 Ammonia Pretreatment

**Figure 1:**
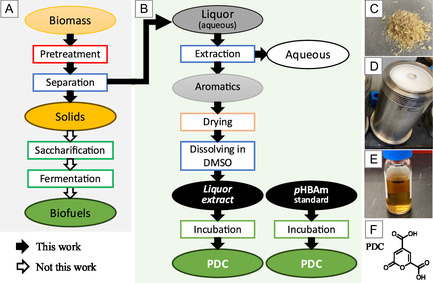
A) Typical procedure of biofuels production from biomass with pretreatment. B) Main procedure of this work: poplar wood chips were pretreated in 18 wt% aqueous ammonia at 145 °C; spent liquor was filtered, collected, and extracted with ethyl acetate. After removing solvent, the extracted organics were redissolved in DMSO‐d_6_ (called *Liquor extract*) for analysis and incubation with *N. aromaticivorans* PDC strain to produce 2‐pyrone‐4,6‐dicarboxylic acid (PDC). C) Poplar wood chips, 5 mm size. D) Stainless steel bombs with Teflon liner. E) *Liquor extract* in DMSO. F) PDC molecular structure, chemical formula C_7_H_4_O_6_, molecular weight 184.10 g mol^−1^.

For the ammonia pretreatment of poplar wood (**Figure** **1** and **Table** **1**, #1‐6), to a 300 mL Teflon liner (Grace Instrument), 20 or 30 g dry poplar wood chips, metal additive, aqueous ammonia (NH_4_OH, 28.0–30.0 wt%), and RO water were added to reach a liquid/solid ratio of 4:1, a final ammonia concentration of 18 wt% in liquid, and designed dose of metal (biomass basis). To investigate the effects of metals on ammonia pretreatment, five different metal additives were added. The Cu(bpy) was prepared from CuSO_4_ and bpy, and used at a final Cu/bpy mole ratio of 1:1 following literature.^[^ ^32^ ^]^ Other metal additives were added as received. The Teflon liner was capped and loaded into a 500 mL stainless‐steel cell (Grace Instrument Company), which was sealed and loaded into a roller oven (Grace Instrument Company). The rollers were turned on for mixing with oven temperature targeted at 145 °C and total time at 3.5 h, which included ≈50 min for heating up and ≈160 min holding at 145 °C. At the end of 3.5 h, the heater automatically shut off and the oven was cooled down overnight with the oven door closed and rollers running.

**Table 1:** Conditions of ammonia treatment and quantified phenolic substrates before and after treatment of poplar wood chips (#1–6), *p*HBA standards (#7–13), and *p*HBAm standards (#14–20).

#	Substrate [g]	Liquid [mL]a)	Metal additive	Metal dose [wt%]b)	Total time [h]c)	Substrate [mM]	*p*HBAm [mM]	*p*HBA [mM]	Phenol [mM]	Total [mM]	Recovery [%]	Phenol Yield [%]	*p*HBAm:*p*HBA:Phenol [LC]	*p*HBAm:*p*HBA:Phenol [NMR]
**Poplar wood chips pretreatment**														
1	30	120	Control	No metal	3.5	23.8d)	6.07e)	1.36	13.0	20.4	86	58f)	29:07:64	28:07:65
2	30	120	Cu(bpy)	0.07	3.5	23.5	3.80	0.50	10.7	15.0	64	47	26:03:71	26:03:71
3	20	80	Pd(OAc)_2_	0.51	3.5	25.3	5.03	0.70	10.4	16.1	64	43	32:04:64	34:05:61
4	20	80	Al_2_O_3_	0.51	3.5	25.4	5.91	0.80	11.1	17.8	70	46	33:04:63	31:05:64
5	20	80	NiCl_2_	0.05	3.5	25.6	4.85	0.59	12.0	17.4	68	49	28:03:69	27:04:69
6	20	80	Mg(OAc)_2_	0.50	3.5	25.6	5.43	0.84	11.9	18.2	71	49	30:04:66	30:04:66
***p*** **HBA decarboxylation tests**													***p*** **HBA:Phenol**	
7	0.08	80	Control	No metal	2.0	6.69	ND	2.84	3.99	6.83	102	104g)	42:58	
8	0.08	80	Control	No metal	3.5	6.59	ND	0.18	5.99	6.17	94	93	03:97	
9	0.08	80	Cu(bpy)	0.07	3.5	6.88	ND	0.18	6.40	6.59	96	96	03:97	
10	0.08	80	Pd(OAc)_2_	0.52	3.5	6.76	ND	0.21	6.12	6.33	94	94	03:97	
11	0.08	80	Al_2_O_3_	0.50	3.5	6.83	ND	0.41	5.89	6.30	92	92	06:94	
12	0.08	80	NiCl_2_	0.05	3.5	6.85	ND	0.35	6.13	6.48	95	94	05:95	
13	0.08	80	Mg(OAc)_2_	0.50	3.5	6.80	ND	0.20	6.26	6.45	95	95	03:97	
***p*** **HBAm stability tests**														
14	0.08	80	Control	No metal	2.0	7.09	6.99	ND	ND	6.99	99	0		
15	0.08	80	Control	No metal	3.5	6.97	7.01	ND	ND	7.21	101	0		
16	0.08	80	Cu(bpy)	0.07	3.5	6.94	6.87	ND	ND	7.08	99	0		
17	0.08	80	Pd(OAc)_2_	0.53	3.5	6.89	7.15	ND	ND	7.10	104	0		
18	0.08	80	Al_2_O_3_	0.44	3.5	6.91	7.04	ND	ND	7.09	102	0		
19	0.08	80	NiCl_2_	0.06	3.5	6.96	7.01	ND	ND	7.27	101	0		
20	0.08	80	Mg(OAc)_2_	0.50	3.5	6.89	6.75	ND	ND	7.11	98	0		

a) 18 wt% NH_4_OH aq. (NH_3_ basis).

b) As pure metal, based on dry biomass.

c) Including 50 min of ramping up from room temperature (≈23 °C) to 145 °C and the time holding at the temperature.

d) For #1–6 only, as *p*HB‐ester (measured as *p*HBA after basic hydrolysis at 90 °C for 90 min) at the beginning of pretreatment in 18 wt% NH_4_OH aq.; the small difference was due to slightly different initial biomass weights.

e) At the end of pretreatment.

f) Phenol molar yield based on released *p*HB‐ester, e.g., 13.0/(23.8‐residual *p*HB‐ester in solids) × 100% = 58%; the residual *p*HB‐ester in solids was 3%–5% of original *p*HB‐ester in wood (Table S1, Supporting Information).

g) Phenol molar yield based on consumed *p*HBA, e.g., 3.99/(6.69‐2.84)×100% = 104%. g = gram, mL = milliliter, wt% = weight percent, h = hour, mM = millimolar, *p*HBAm = *p*‐hydroxybenzamide, *p*HBA = *p*‐hydroxybenzoic acid, ND = Not detected.

After cooling down to room temperature, the slurry was transferred into a 500 mL stainless‐steel cell with a 2500 mesh (5 μm) membrane (USA Lab, Buna‐n gasket with mesh made of stainless steel 304, diameter 3 inches). Pressurized air was applied from the top of the stainless‐steel cell to drive the solid and liquid separation. The spent liquor was collected for later use.

Same operations were carried out to evaluate the transformations of *p*HBA (Table 1, #7–13) and *p*HBAm (#14–20) in the ammonia pretreatment environment. The generated liquid samples were neutralized with 4 N H_2_SO_4_ aq. and filtered for liquid chromatography–mass spectrometry (LC–MS) analysis.

### 2.3 Liquid–Liquid Extraction, LC–MS, and LC Fractionation

The spent liquor (pH ≈11.5) was first extracted by two volumes of ethyl acetate to remove organic compounds that were extractable under high alkaline conditions. After collecting the ethyl acetate layer, the residual spent liquor was acidified to pH 1–1.5 with 72 wt% sulfuric acid (H_2_SO_4_) and extracted a second time by two volumes of fresh ethyl acetate to remove organic compounds that are more extractable under acidic conditions (e.g., *p*HBA). The acidic and basic ethyl acetate extracts were combined and dried on a Heidolph rotary evaporator for ≈45 min and a freeze dryer overnight, the residual was so called *liquor extract*. The *liquor extract* was dissolved in DMSO‐d_6_ for nuclear magnetic resonance (NMR) spectroscopy, LC–MS analysis, chromatographic fractionation, and microbial conversion to PDC. The *liquor extract* in DMSO‐d_6_ was diluted with DMSO as needed.

LC–MS analysis was conducted on a Shimadzu Prominence HPLC system with a Phenomenex Kinetex PFP analytical column (5 μm, 100 Å, 250 × 4.60 mm), LCMS‐8040 mass spectrometer, and a PEAK Scientific Genius nitrogen generator. The chromatogram, PDA absorption (250–500 nm), and mass spectra were collected for each run. The mobile phase was 0.1 v% formic acid in Mili‐Q water (phase A) and 0.1 v% formic acid in 3:1 v/v acetonitrile:methanol (phase B). A linear 15‐minute gradient program was used to control the mobile phase composition: phase B was 1% at 0–0.5 min, 14% at 1 min, 16% at 5 min, 80% at 8 min, 95% at 10.5–11.5 min, and back to 1% at 13–15 min to reset the column. The injection volume was 1–30 μL as necessary, which was proportional to PDA area for the same peak when the detector was not saturated. The *p*HBAm, *p*HBA, and phenol standards were dissolved in methanol for LC–MS analysis, while their standard curves were established using the peak areas of PDA absorption and corresponding concentrations (mM), resulting in the following formulas,

(1) $$C_{p\text{HBAm}}\;=\;1.442E-06\;\times \;A_{p\text{HBAm}@250nm}$$

(2) $$C_{p\text{HBA}}\;=\;1.174E-06\;\times \;A_{p\text{HBA}@250nm}$$

(3) $$C_{Phenol}\;=\;1.137E-05\;\times \;A_{Phenol@270nm}$$

where *C* denotes concentrations (mM) and *A* indicates PDA absorption areas of *p*HBAm or *p*HBA at 250 nm and of phenol at 270 nm.

LC fractionation of the *liquor extract* in DMSO was conducted on a Shimadzu Prominence HPLC system with a Phenomenex Kinetex F5 semi‐preparation column (5 μm, 100 Å, 250 × 10 mm) and a Shimadzu SPD‐M20A preparatory flow cell. Mobile phase was the same as LC–MS. A linear 45‐minute gradient program was used to control the mobile phase composition: phase B was 1% at 0‐5 min, 16% at 20 min, 95% at 34–37 min, and back to 1% at 40–45 min to reset the column. The injection volume was 50 or 100 μL for each run to balance the best separation and highest injection volume. Fractions separated by the semi‐preparation column were collected after the flow cell, which were dried and re‐dissolved in DMSO‐d_6_ for NMR and LC–MS analysis.

### 2.4 NMR Spectroscopy

NMR spectra of the *liquor extract* samples and the reference standards (*p*HBAm, *p*HBA, phenol, bpy) were acquired using DMSO‐d_6_ as the solvent on a NEO 700 MHz spectrometer (Bruker Corp., Billerica, MA, USA) with a cryogenically cooled 5 mm QCI ^1^ H/^31^ P/^13^C/^15^ N cryoprobe with inverse geometry (proton coils closer to the sample).^[^ ^59^, ^60^, ^61^ ^]^ The ^1^ H–^13^C correlation experiment was an adiabatic HSQC experiment (Bruker standard pulse sequence “hsqcetgpsisp2.2”; phase‐sensitive gradient‐edited‐2D heteronuclear single quantum coherence (HSQC) using adiabatic pulses for inversion and refocusing),^[^ ^62^ ^]^ which was carried out using the following parameters: acquired from 11.65 to −0.66 ppm in F2 (^1^ H) with 3,448 data points (acquisition time, 200 ms) and 215 to−5 ppm in F1 (^13^C) with 618 increments (F1 acquisition time, 8 ms) of 8 scans with a 1 s interscan delay; the d_24_ delay was set to 0.89 ms (1/8 J, J = 140 Hz). Data were processed with the Bruker TopSpin 4.4.0 software (Mac version), using typical matched Gaussian apodization (LB = −0.1, GB = 0.001) in F2 and squared cosine‐bell in F1 (without using linear prediction). The central solvent peak (DMSO‐d_6_ δ_H_ 2.49 δ_C_ 39.50 ppm) was used as reference. The area integrals of *p*HBAm, *p*HBA, and phenol in the HSQC spectra were used to determine their mole ratios, as summarized in Table 1.

### 2.5 *pHB‐Ester* in Poplar Wood and Solids after Pretreatment

The ester‐linked *p*‐hydroxybenzoate (*p*HB‐ester) in poplar wood and the solids after ammonia pretreatment were determined following the literature.^[^ ^20^, ^24^ ^]^ In brief, wood powder (5 mg) or solids after pretreatment (15 mg) were treated with 1 mL 2 M NaOH aq. in 2 mL vial at 90 °C for 90 min in sand bath. The reaction was stopped by soaking the vials in ice water. After centrifugation at 19 000 rpm for 3 min, the supernatant was filtered (0.45 μm nylon filter) and analyzed by LC–MS to determine the molar amount of *p*HBA, which was used as the mole amount of *p*HB‐ester (*N*_*pHB‐ester*_) in poplar wood or solids after pretreatment. A theoretical concentration of *p*HB‐ester (*C*_*pHB‐ester*_, in mM), in reaction liquid was calculated according to the following formula,

(4) $$C_{pHB\text{-}ester}\;=\;N_{pHB\text{-}ester}\;/\;V_{liquid}$$

where *N*_*pHB‐ester*_ is the amount of *p*HB‐ester (mmol) in poplar wood chips or treated solids, and *V*_*liquid*_ is the volume of total liquid (liter) of the ammonia pretreatment.

The recovery of *p*HB‐ester (Table 1, #1–6) was then calculated according to following formula,

(5) $$Recovery\;\left(\%\right)\;=\;\left(C_{pHBAm}\;+\;C_{pHBA}\;+\;C_{Phenol}\right)\;/\;\left(C_{pHB\text{-}ester\_t0}\;–\;C_{pHB\text{-}ester\_t1}\right)\;\times \;100\%$$

where *C*_*pHBAm*_ *+ C*_*pHBA*_ *+ C*_*Phenol*_ (in mM) is the detected total concentration of the three phenolics in spent liquor after ammonia pretreatment, *C*_*pHB‐ester‐t0*_ and *C*_*pHB‐ester_t1*_ are the theoretical concentrations of *p*HB‐ester at the beginning and end of the ammonia pretreatment as determined according to formula (4).

Similarly, the recovery of *p*HBA (Table 1, #7–13) or *p*HBAm (#8–20) was calculated according to the following formula,

(6) $$Recovery\;\left(\%\right)\;=\;\left(C_{pHBAm}\;+\;C_{pHBA}\;+\;C_{Phenol}\right)\;/\;C_{substrate\_t0}\;\times \;100\%$$

where *C*_*substrate_t0*_ is the theoretical concentration of *p*HBA or *p*HBAm at the beginning of the ammonia treatment.

### 2.6 PDC Production from Extracted Phenolics

PDC production was conducted following previous studies.^[^ ^55^, ^56^, ^57^ ^]^ To each sterile Erlenmeyer flask, 200 μL of aromatic‐containing sample, 8.8 mL of SMB media,^[^ ^58^ ^]^ and 1 mL culture of *N. aromaticivorans* PDC strain were added under sterile conditions. After mixing, a 0.6 mL aliquot was immediately taken and filtered for the measurements of initial concentrations (*t*_0_). Incubation was conducted in a shaker (Innova 2300) at 30 °C and 200 pm. At 24 h, another 0.6 mL aliquot was collected and filtered for 24 hr concentration measurements (*t*_24_). The collected samples were stored in freezer before analysis. Cell density was assessed with a Klett–Summerson Photoelectric Colorimeter at the time of inoculation and after the 24‐hr incubation.

The phenolics and PDC at the beginning and end of incubation were determined by LC–MS. PDC standard was dissolved in DMSO for LC–MS analysis, to establish its standard curve using peak areas of PDA absorption at 313 nm,^[^ ^57^ ^]^ resulting in following formula,

(7) $$C_{PDC}\;=\;2.211E-06\;\times \;A_{PDC@313nm}$$

where *C*_*PDC*_ is the PDC concentration (mM) and *A*_*PDC*_ is the PDC peak area of the PDA absorption at 313 nm.

The PDC molar yield (*Y*_*PDC*_) based on consumed [*p*HBAm+*p*HBA] was calculated according to the following formula,

(8) $$Y_{PDC}\;\left(\%\right)\;=\;C_{PDC\_t24}\;/\;\left(C_{pHBAm\_t0}\;+\;C_{pHBA\_t0}\;-\;C_{pHBAm\_t24}\;-\;C_{pHBA\_t24}\right)\;\times \;100\%$$

The PDC molar yield (*Y*_*PDC*_) based on initial [*p*HBAm+*p*HBA] was calculated according to the following formula,

(9) $$Y_{PDC}\;\left(\%\right)\;=\;C_{PDC\_t24}\;/\;\left(C_{pHBAm\_t0}\;+\;C_{pHBA\_t0}\right)\;\times \;100\%$$

where *C*_*PDC_t24*_ *, C*_*pHBAm_t24*_, and *C*_*pHBA_t24*_ are the concentrations (mM) of PDC, *p*HBAm, and *p*HBA at 24 h of incubation, and *C*_*pHBAm_t0*_ and *C*_*pHBA_t0*_ are the concentrations (mM) of *p*HBAm and *p*HBA at the beginning of incubation.

## 3 Results and Discussions

### 3.1 Characterization of Liquor Extract

#### 3.1.1 Composition Overview via NMR

**Figure 2:**
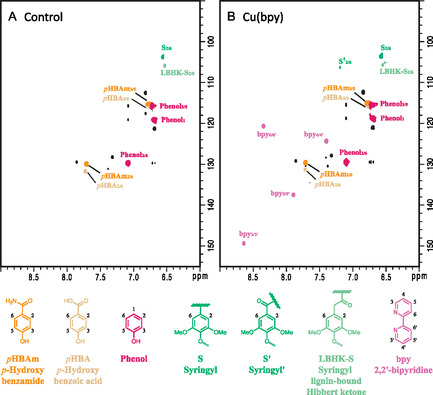
HSQC NMR spectra of representative *liquor extract* samples. A) Control, no metal additive during ammonia pretreatment. B) Cu(bpy) added during pretreatment. For NMR spectra of all *liquor extract* samples, please see Figure S1, Supporting Information.

The *liquor extract* samples were generated via solvent extraction of spent liquor from ammonia pretreatment. The HSQC NMR spectra of all *liquor extract* samples were similar: a major peak of acetamide (CH_3_CONH_2_, δ_H_ 1.79 δ_C_ 22.5 ppm) and few small peaks around it; and strong signals in aromatic region, as shown in Figure S1, Supporting Information. The peaks in aromatic region were assigned with details, and two representative spectra are shown in **Figure** **2**. The main peaks were assigned according to the spectra of standards, i.e., *p*‐hydroxybenzamide (*p*HBAm_2/6_ δ_H_ 7.72 δ_C_ 129.3 ppm, *p*HBAm_3/5_ δ_H_ 6.77 δ_C_ 114.5 ppm), *p*‐hydroxybenzoic acid (*p*HBA_2/6_ δ_H_ 7.78 δ_C_ 131.3 ppm, *p*HBA_3/5_ δ_H_ 6.81 δ_C_ 114.9 ppm), phenol (Phenol_1_ δ_H_ 6.75 δ_C_ 118.6 ppm, Phenol_2/6_ δ_H_ 7.14 δ_C_ 129.2 ppm, Phenol_3/5_ δ_H_ 6.73 δ_C_ 115.0 ppm), and 2,2′‐bipyridine (bpy_3/3'_ δ_H_ 8.68 δ_C_ 149.1 ppm, bpy_4/4'_ δ_H_ 7.44 δ_C_ 124.0 ppm, bpy_5/5'_ δ_H_ 7.93 δ_C_ 137.1 ppm, bpy_6/6'_ δ_H_ 8.38 δ_C_ 120.2 ppm). The syringyl structures (S_2/6_ δ_H_ 6.57 δ_C_ 103.6, S'_2/6_ δ_H_ 7.19 δ_C_ 106.3, LBHK‐S_2/6_ δ_H_ 6.55 δ_C_ 105.7) were assigned according to literature.^[^ ^22^, ^59^, ^63^, ^64^ ^]^ There were also un‐identified signals in the aromatic region of the HSQC NMR (black lines).

#### 3.1.2 Phenolics Based on LC and NMR

**Figure 3:**
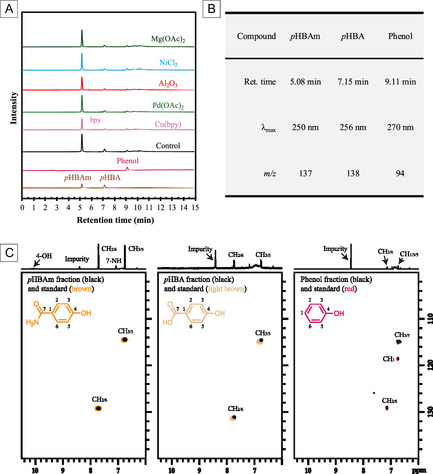
Characterization of *liquor extract* samples and main fractions. A) LC chromatograms (254 nm) of *liquor extract* samples and standards of *p*HBAm, *p*HBA, and phenol. Abbreviations: *p*HBAm, *p*‐hydroxybenzamide; *p*HBA, *p*‐hydroxybenzoic acid; bpy, 2,2′‐bipyridine; OAc, acetate. B) Summary of the main peaks on LC–MS chromatograms and their properties. λ_max_, wavelength at which the compound has the strongest photon absorption (LC–MS did not collect < 250 nm). C) HSQC NMR (in box) and ^1^ H NMR (above box) spectra of the fractions of *p*HBAm, *p*HBA, and phenol, and their standards.

To further identify the main phenolics, the *liquor extract* samples were analyzed by LC–MS. **Figure** **3** A shows the LC chromatograms (λ = 254 nm) of standards (*p*HBAm, *p*HBA, and phenol) and all *liquor extract* samples. As summarized in Figure 3B, the *liquor extract* samples contained a large peak that eluted at 5.08 min, which matched the retention time, mass‐to‐charge ratio (*m/z* = 137), and UV absorption profile (λ_max_ = 250 nm) of the *p*HBAm standard. The peak at 7.15 min matched the properties of *p*HBA standard (*m/z* = 138, λ_max_ = 256 nm), and the peak at 9.11 min matched the properties of phenol standard (*m/z* = 94, λ_max_ = 270 nm). For the sample with Cu(bpy), the 2,2'‐bipyridine (bpy) eluted as a broad peak at 6.3 min. There were also weak peaks around 9.3–10.5 min.

Fractionation of the *liquor extract* was conducted on a Kinetex F5 semipreparation column to verify the identity of these components. Each fraction was collected, dried, and redissolved in DMSO‐d_6_ for LC–MS (Figure S2, Supporting Information) and NMR analysis. Figure 3C shows the NMR ^1^ H (above box) and ^1^ H–^13^C HSQC (in box) spectra of the collected *p*HBAm, *p*HBA, and phenol fractions, which matched exactly with the spectra of their standards.

The concentrations of *p*HBAm, *p*HBA, and phenol in the *liquor extract* samples were determined using external calibration curves, and their corresponding concentrations in original spent liquor are summarized in Table 1 (#1–6). The *p*HBAm concentration (in mM) at the end of ammonia pretreatment decreased by 37%, 17%, 3%, 20%, and 10% in the presence of Cu(byp) (#2), Pd(OAc)_2_ (#3), Al_2_O_3_ (#4), NiCl_2_ (#5), and Mg(OAc)_2_ (#6) versus the control (#1, 6.07 mM), respectively. The *p*HBA concentration decreased by 64%, 48%, 41%, 57%, and 38% for #2–6 versus #1 (1.36 mM). And phenol concentration decreased by 18%, 20%, 14%, 8%, and 8% for #2–6 versus #1 (13.0 mM).

To determine if *p*HBA produced *p*HBAm and phenol, and if metal additives promoted the synthesis and/or degradation of *p*HBAm, *p*HBA, and phenol, the ammonia pretreatment of only *p*HBA or *p*HBAm control and with metal additives were studied and their results were summarized in Table 1 (#7–20). The results showed that *p*HBA was converted to phenol almost quantitatively (yield 92%–104%), but *p*HBAm was not produced from *p*HBA, indicating decarboxylation but not amination happened to *p*HBA under ammonia pretreatment at temperature used in this study (≤145 °C). The total recovery of *p*HBA substrate at longer reaction time (#8) was lower than that at shorter reaction time (#7), indicating possible degradation, whereas *p*HBAm is stable at longer reaction time. At longer reaction time, *p*HBA was converted to phenol more completely (#7 vs #8–13). At the same reaction time for ammonia pretreatment of *p*HBA (#8–13), metal additives did not show much effect. At the same reaction time for ammonia pretreatment of *p*HBAm (#15–20), metal additives did not show any effect either. Thus, we can conclude that *p*HBAm and *p*HBA were produced directly from the ester‐linked *p*‐hydroxybenzoate (*p*HB‐ester) in poplar wood during ammonia pretreatment, then phenol was produced from *p*HBA via decarboxylation. Additionally, slow degradation of *p*HBA can happen under the conditions in this study.

To determine the recovery of *p*HB‐ester and phenol yield, the original *p*HB‐ester in poplar wood and the residual *p*HB‐ester in solids after ammonia pretreatment were all measured as *p*HBA via LC–MS after hydrolysis in 2 M NaOH aq. at 90 °C for 90 min. As summarized in Table S1, Supporting Information, raw poplar wood had 0.110 mmol g^−1^ *p*HB‐ester, which equals to 1.5 wt% of poplar wood (as *p*HBA). The residual *p*HB‐ester (mmol/g biomass) in the solids after ammonia pretreatment was only 3%–5% of the original in un‐treated wood. The recovery of original *p*HB‐ester after ammonia pretreatment of poplar wood was calculated according to formula (4), (5) for all samples (Table 1, #1–6). The *p*HB‐ester recovery was 86 mol% for the control (#1), indicating possible side reactions happened to the *p*HB‐ester besides the degradation of the phenolics, because the phenolics loss caused by degradation would be smaller (≤8%, as discussed above) than the current loss (14%). The *p*HB‐ester recovery was lower for #2–6 (64%–71%), indicating metal additives might have promoted the side reactions, because metal additives would not cause any extra loss of these phenolics as discussed above. And same thing happened to the phenol yields, i.e., the phenol yield of 58% for control (#1) was due to both possible side reactions and phenol degradation, while the phenol yields of 43%–49% indicated that metal additives might have promoted side reactions of *p*HB‐ester degradation.

The phenolic mole ratio (*p*HBAm:*p*HBA:Phenol) was determined by both LC–MS, and HSQC NMR (Table 1). The ratios from HSQC NMR matched exactly with the ratios from LC–MS, indicating HSQC integral can be very accurate at determining compound ratio. The phenolic ratios of #2 and #5 were slightly different from others, indicating Cu(bpy) and NiCl_2_ might have slightly promoted the *p*HBA production over *p*HBAm, while Al_2_O_3_ might have slightly promoted the *p*HBAm production over *p*HBA. The *p*HBAm/*p*HBA ratio was 82:18 for #1 and 88:12 for #2–6, both lower than 99:1 from previous study.^[^ ^24^ ^]^ This previous study showed when poplar wood was pretreated with ammonia (NH_4_OH), *p*HBAm and *p*HBA were produced from the *p*HB‐ester in poplar lignin, during which reaction temperature played an role, i.e., the *p*HBAm/*p*HBA ratio was around 90:10, 50:50, and 99:1 at the temperature of 25–40, 100, and 140 °C, respectively at 24 h in 29 wt% NH_4_OH aq.^[^ ^24^ ^]^ The *p*HBAm/*p*HBA ratio of present study did not reach 99:1, which was most likely due to a combination of two factors: 1) the slower temperature ramp rate in the roller oven of current study versus the preheated sand bath used in previous study,^[^ ^24^ ^]^ means longer reaction time below 100 °C of current study where hydrolysis of the *p*HB‐ester to produce *p*HBA was favored over amination of the *p*HB‐ester to *p*HBAm. 2) The overall shorter reaction time in the current study (vs 24 h in literature)^[^ ^24^ ^]^ reduced the amount of *p*HBA degradation to phenol via decarboxylation, resulting in a higher *p*HBA ratio.

### 3.2 PDC Production

#### 3.2.1 PDC Production from *Liquor Extract*

It is known that *N. aromaticivorans* PDC strain can efficiently transform *p*HBA to PDC,^[^ ^55^, ^56^, ^57^ ^]^ but to date, there are no reports of *N. aromaticivorans* being able to use *p*HBAm as an organic substrate. Analysis of *N. aromaticivorans* genome indicates the presence of four proteins (Saro_3057, Saro_3602, Saro_3815, and Saro_0605) which were putatively annotated as amidases, indicating that *N. aromaticivorans* might be to use transform *p*HBAm to *p*HBA.^[^ ^65^, ^66^ ^]^ There are limited studies of aromatic degrading amidases converting *p*HBAm to *p*HBA, with in vitro activity being demonstrated in *Rhodococcus rhodochrous* ^[^ ^67^ ^]^ and *Pseudonocardia thermophila*.^[^ ^68^ ^]^ Amidase activity in *Rhodopseudomonas palustris* grown on ammonia fiber expansion (AFEX)‐treated corn stover hydrolysate exhibited strong aromatic degrading amidases activity with feruloylamide and *p*‐coumaroylamide, but comparatively very weak activity with *p*HBAm.^[^ ^69^ ^]^ From gene annotations, it is predicted that if *N. aromaticivorans* degrades phenol, the transformation would occur via a catechol pathway that would not produce PDC as an intermediate.^[^ ^44^ ^]^ Thus, we investigated the transformation of these phenolics to PDC by adding *liquor extract* aliquots to *N. aromaticivorans* PDC strain cultures growing on SMB plus glucose. Figure S3, Supporting Information shows the representative LC chromatograms of liquid samples at the beginning (*t*_*0*_) and 24 h (*t*_*24*_) of incubation with the peaks corresponding to PDC (retention time 5.0 min), *p*HBAm (5.2 min), bpy (6.4 min), *p*HBA (7.1 min), and phenol (9.1 min). The retention times were slightly different from Figure 3A, which might be due to the different pH, salt, and concentration of the incubation media. From the beginning (*t*_*0*_) to 24 h incubation (*t*_*24*_), the extracellular *p*HBAm and *p*HBA were mostly consumed, PDC accumulated in the media, while the peaks of bpy and phenol stayed essentially the same, indicating biotransformation of *p*HBAm and *p*HBA and production of PDC from them.

**Figure 4:**
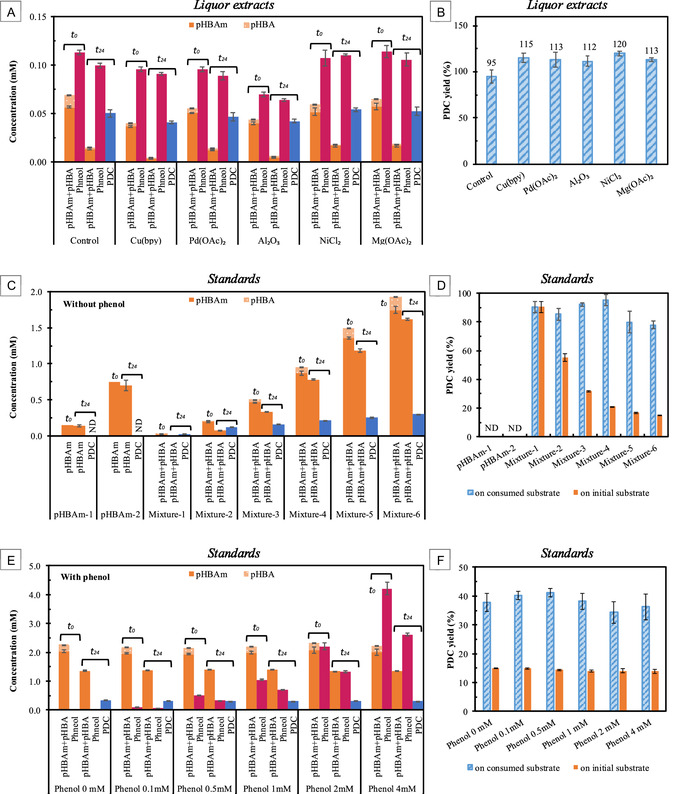
PDC (2‐pyrone‐4,6‐dicarboxylic acid) production via biotransformation. A) PDC production from *liquor extracts* from ammonia pretreatment of poplar wood without (Control) and with metal additives (Cu(bpy), Pd(OAc)_2_, Al_2_O_3_, NiCl_3_, Mg(OAc)_2_). B) PDC mole yields based on consumed [*p*HBAm + *p*HBA] from (A). C) PDC production from *p*HBAm standards and mixed [*p*HBAm+*p*HBA] (10:1 mol mol^−1^) standards without phenol. D) PDC mole yields based on consumed or initial [*p*HBAm+*p*HBA] from (C). E) PDC production from mixed [*p*HBAm+*p*HBA] (10:1 mol/mol) standards with various phenol levels (up to 4 mM). F) PDC mole yields based on consumed or initial [*p*HBAm+*p*HBA] from (E). t_0_, at the beginning of microbial incubation; t_24_, at 24 h of incubation. Error bar, standard error of the mean (SEM), N = 3.

The concentrations of *p*HBAm, *p*HBA, phenol, and PDC at the beginning (*t*_*0*_) and 24 h (*t*_*24*_) of incubation were determined by LC according to formula (1), (2), (3), (7), and summarized in **Figure** **4**A. In general, there were about 0.06 mM of total *p*HBAm plus *p*HBA ([*p*HBAm*+p*HBA]) and about 0.09 mM of phenol at the beginning of incubation, then about 0.05 mM PDC was produced with some residual *p*HBAm and *p*HBA at 24 h incubation, while there might be a smaller decrease in phenol concentrations. The PDC molar yields from the total consumed *p*HBAm and *p*HBA were calculated according to formula (8) and shown in Figure 4B. The control culture had a PDC yield of 95%, while samples with metal additives had yields around 115%, indicating phenolics other than *p*HBAm and *p*HBA were converted to PDC. These un‐identified phenolics could be the products of possible side reactions catalyzed by metal additives during ammonia pretreatment, as discussed above, which also appeared in HSQC NMR spectra of similar samples.

#### 3.2.2 PDC Production from *PHBAm* Standard

Since the production of PDC from *p*HBAm and other phenolic amides using *N. aromaticivorans* has never been reported before, we tested *p*HBAm standards as the phenolic substrate, to evaluate whether PDC was produced (Figure 4C–F). When *p*HBAm was used as the sole phenolic substrate, no PDC was detected after 24 h incubation and the *p*HBAm concentration stayed essentially the same (Figure 4C). Given that the experiments with *liquor extract* suggested *p*HBAm transformation to PDC (Figure 4A), we hypothesized that *p*HBAm only does not induce the PDC production pathways in *N. aromaticivorans*. Considering that all the *liquor extract* samples contained *p*HBA and phenol, their effects on PDC production from *p*HBAm were tested.

First, a small amount of *p*HBA was added to the *p*HBAm substrate (*p*HBA/*p*HBAm ratio = 1:10) since *p*HBA can be readily transformed to PDC.^[^ ^55^, ^56^, ^57^, ^58^ ^]^ At various initial [*p*HBAm *+ p*HBA (10:1)] concentrations of 0.03–2 mM, successful PDC production from *p*HBAm was observed in all samples. At 24 h of incubation, the PDC concentrations were 150%–320% of initial *p*HBA concentrations, indicating PDC was produced from *p*HBAm (Figure 4C). At 48 and 96 h of incubation, the PDC concentrations kept increasing (Figure S4A, Supporting Information) even though there was no *p*HBA. When the PDC produced from *p*HBA is not considered, the estimated PDC produced from *p*HBAm ([PDC–*p*HBA]) was above zero and kept increasing at 48 and 96 h of incubation (Figure S4B, Supporting Information). These tests confirmed that *p*HBAm can be converted to PDC using *N. aromaticivorans* PDC strain, though it requires another phenolic compound to activate the needed microbial pathways. At 24 h incubation, the PDC molar yields based on consumed [*p*HBAm*+p*HBA] were ≈90% for samples with initial [*p*HBAm*+p*HBA] concentrations of 0.03–1 mM, reflecting that most of the *p*HBAm*+p*HBA was consumed during the incubation period. At higher initial concentrations, even though more PDC was produced (up to 0.30 mM, Figure 4C), the PDC yield based on the initial [*p*HBAm*+p*HBA] decreased (Figure 4D), consistent with not all the *p*HBAm*+p*HBA being transformed during the incubation period of these experiments.

To test the effects of phenol, a gradient series of phenolic assay media was prepared with 0, 0.1, 0.5, 1, 2, and 4 mM phenol in 2 mM [*p*HBAm*+p*HBA] for incubation. After 24 h of incubation with *N. aromaticivorans* PDC strain, similar amounts of PDC (0.32 mM, Figure 4E) were produced across the phenol levels, which was consistent with the previous incubation on 2 mM initial [*p*HBAm*+p*HBA] without phenol present (Figure 4C, Mixture‐6). The PDC molar yields based on initial [*p*HBAm*+p*HBA] were ≈14% (Figure 4F), which was similar to the 15% yield from the previous incubation on 2 mM initial [*p*HBAm* + p*HBA] without phenol (Figure 4D). This indicates that the PDC was produced from [*p*HBAm *+ p*HBA] but not phenol. The PDC molar yields based on consumed [*p*HBAm *+ p*HBA] were around 38% in this experiment, which were found to be because of extra *p*HBAm loss compared to the experiments without phenol addition (Figure 4C). Additionally, phenol recoveries at 24 h were ≈64% for all samples at various initial phenol concentrations (Figure S4D, Supporting Information), indicating phenol loss during the operations. From these experiments, we can conclude that phenol (up to 4 mM) was not converted to PDC and was not inhibitory to PDC production from [*p*HBAm + *p*HBA] using *N. aromaticivorans*.

## 4 Conclusion

This study characterized the phenolics in spent liquor from aqueous ammonia pretreatment of poplar wood, investigated the impact metal additives had on the formation of phenolics during the pretreatment, and explored the valorization of them via biotransformation to reduce the organics in spent liquor.

**Figure 5:**
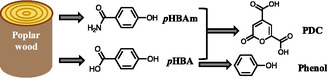
Pathways of producing valuable chemicals from poplar wood via ammonia pretreatment and biotransformation with *N. aromaticivorans* PDC strain.

The main phenolics in spent liquor were determined to be phenol, *p*‐hydroxybenzamide (*p*HBAm), and *p*‐hydroxybenzoic acid (*p*HBA) by fractionation, LC–MS and NMR. The *p*HBAm and *p*HBA were produced from the ester‐linked *p*‐hydroxybenzoate (*p*HB‐ester) in natural lignin of poplar wood, then phenol was produced from *p*HBA via decarboxylation under alkaline conditions, while *p*HBAm was stable (**Figure** **5**). Compared to the control, metal additives reduced the phenolic recovery and phenol yield, likely due to that metal additives might have promoted the side reactions of *p*HB‐ester degradation to produce un‐identified phenolics.

The extracted phenolics, including *p*HBAm and *p*HBA (with phenol present), were successfully converted into PDC using *N. aromaticivorans* PDC strain, indicating PDC production from *p*HBAm was possible, which is the first time we observe *N. aromaticivorans* can make PDC from a phenolic amide. Microbial incubation with *p*HBAm as the sole phenolic compound showed that PDC cannot be made. When a small amount of *p*HBA was added to the *p*HBAm substrate, *N. aromaticivorans* PDC strain was able to convert both compounds to PDC at high yield. When phenol was added to the medium, PDC production was not affected, indicating phenol was not a substrate for PDC production and did not inhibit PDC production. These tests indicate that *N. aromaticivorans* PDC strain can convert *p*HBAm to PDC, but the pathways in by need to be activated by another compound like *p*HBA. This is the first report on making PDC from phenolic amide using *N. aromaticivorans*, supporting the presence of native aromatic degrading amidases in *N. aromaticivorans* and adds to the growing body of knowledge of the versatility of *N. aromaticivorans* as a microbial chassis for the conversion of plant‐derived phenolic compounds to useful products. Future in vitro and in vivo experiments are needed to elucidate the role the putative amidases, the aromatic amidase activation pathways, and identify the proteins involved in converting *p*HBAm to PDC.

Recently, the catechol pathway in *N. aromaticivorans* has been engineered for production of *cis,cis*‐muconic acid from *p*HBA,^[^ ^44^ ^]^ and therefore, it may be possible to engineer *N. aromaticivorans* to degrade phenol via the catechol pathway and thus to potentially produce *cis,cis*‐muconic acid from all three major monoaromatics identified in this study.

## Conflict of Interest

The authors declare no conflict of interest.

## Supporting information

Supplementary Material

## Data Availability

The data that support the findings of this study are available in the supplementary material of this article.
