# Supplementary material for: Biotransformation of Phenolics in Spent Liquor from Aqueous Ammonia Pretreatment
Source: ChemSusChem. 2025 Sep 10;18(22):e202500881. doi: 10.1002/cssc.202500881 (PMC12642969; doi:10.1002/cssc.202500881)
Supplement: Supplementary file 1 — Supplementary Material [file CSSC-18-e202500881-s001.pdf]

Biotransformation of Phenolics in Spent Liquor from Aqueous Ammonia Pretreatment

Shengfei Zhou,[a] Maximiliano García Mancilla,[a,b] Jordi Francis Clar,[a] Troy M. Runge,[a,c] Timothy J. Donohue,[a,b] Daniel R. Noguera,[a,d] and Steven D. Karlen*[a]

[a] Great Lakes Bioenergy Research Center, Wisconsin Energy Institute, University of Wisconsin-Madison, Madison, WI 53706, USA
Corresponding author: skarlen@wisc.edu
[b] Department of Bacteriology, University of Wisconsin-Madison, Madison, WI 53706, USA
[c] Department of Biological Systems Engineering, University of Wisconsin-Madison, Madison, WI 53706, USA
[d] Department of Civil and Environmental Engineering, University of Wisconsin-Madison, Madison, WI 53706, USA

Supporting Information

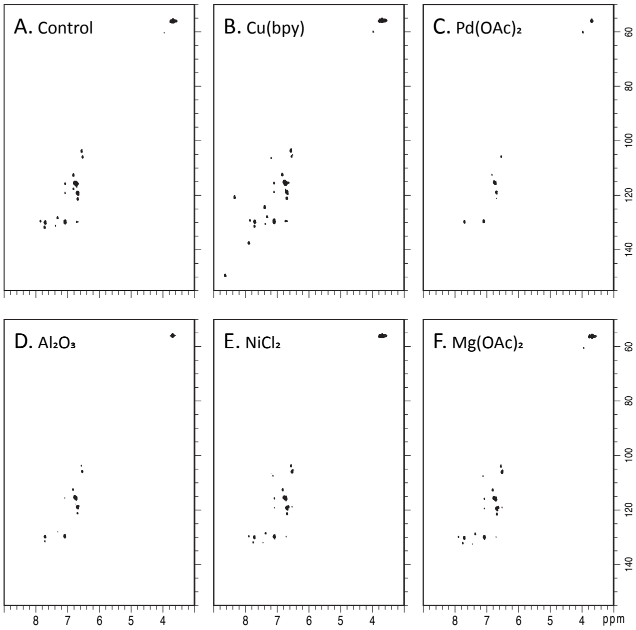

Figure S1. HSQC NMR spectra of *liquor extract* samples at ranges of δ_H 3–9 ppm, δ_C 50–155 ppm. (A) Control, no metal additive during pretreatment. (B) Cu(bpy) added during pretreatment. (C) $Pd(OAc)_2$ added. (D) Al_2O_3 added. (E) $NiCl_2$ added. (F) $Mg(OAc)_2$.

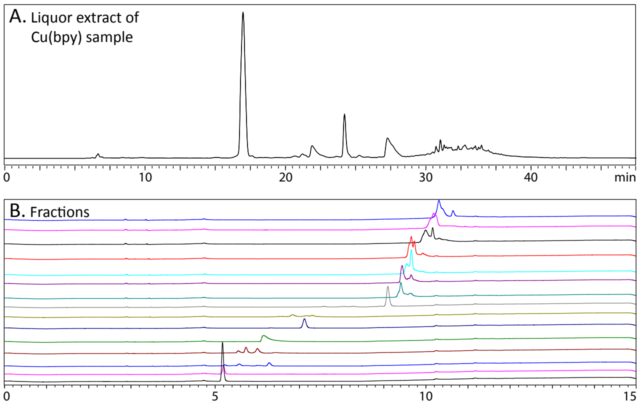

Figure S2. An example of fractionation of *liquor extract* on LC system with semi-preparation column. (A) *Liquor extract* of Cu(bpy) sample, detected on LC system with a Phenomenex Kinetex F5 semi-preparation column in 45 min. Each fraction was collected after the detector, then analyzed on LC system with a Phenomenex Kinetex PFP analytical column in 15 min, resulting in (B) fractions of this *liquor extract* sample. The retention times of each fraction in (A) and (B) were not the same because the two LC systems used different columns and total times were different though their mobile phase gradients were similar; the fractions were in same chronological order in both (A) and (B).

Table S1. *p*HB-ester in poplar wood before and after pretreatment.

#	*p*HB-ester (mmol/g solids) [a]	Residual (%) [b]
Poplar wood NM6	0.110 ± 0.002 [c]	
1	0.0060 ± 0.0001	5
2	0.0028 ± 0.0000	3
3	0.0060 ± 0.0002	5
4	0.0057 ± 0.0003	5
5	0.0044 ± 0.0002	4
6	0.0056 ± 0.0002	5

[a] Average ± standard error of the mean (SEM, N=6). [b] Based on original *p*HB-ester in poplar wood (mmol/g biomass). [c] Equals to 1.5 wt% of poplar wood (as *p*HBA).

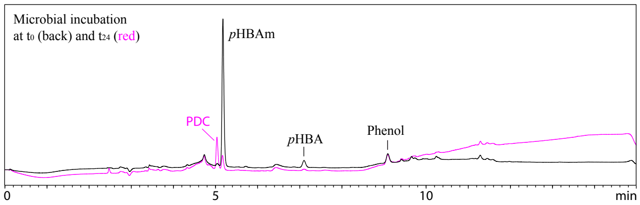

Figure S3. Representative LC spectra (250 nm) of samples at the beginning (t_0) and 24 h (t_{24}) of microbial incubation. Detector channel: PDA 250 nm for both spectra. Black t_0: 5.2 and 7.1 minute for *p*HBAm and *p*HBA. Red t_{24}: 5.0 minute for PDC. Note: PDC λ_{max} = 313 nm.

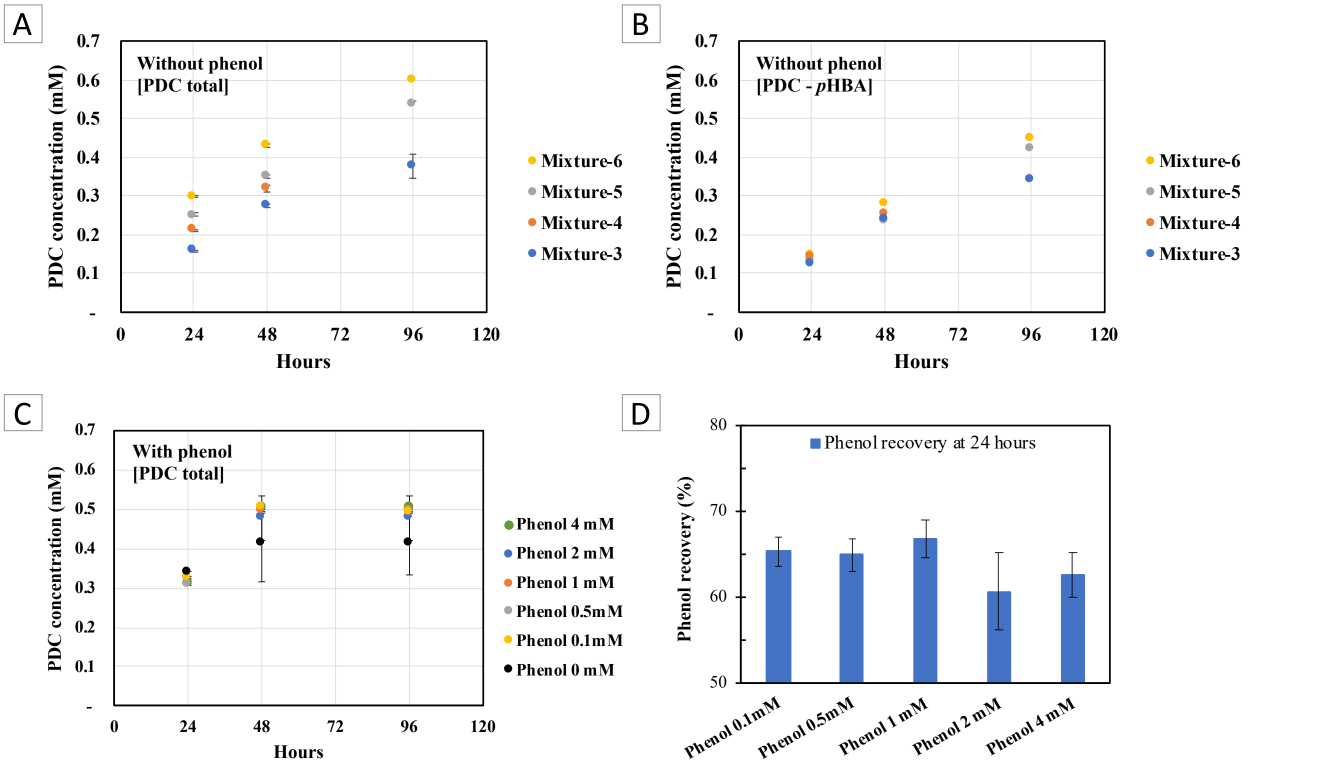

Figure S4. PDC production from [*p*HBAm+*p*HBA] standards via biotransformation. (A) PDC concentrations produced from [*p*HBAm+*p*HBA], without phenol. (B) Average PDC concentrations produced from *p*HBAm by removing the PDC produced from *p*HBA, without phenol. (C) PDC concentrations produced from 2 mM initial [*p*HBAm+*p*HBA], with phenol concentrations of 0, 0.1, 0.5, 1, 2 and 4 mM at incubation. (D) Phenol recovery at 24 hours of incubation. Error bar, standard error of the mean (SEM), N=3.
